# Learning generative models of molecular dynamics

**DOI:** 10.1186/1471-2164-13-S1-S5

**Published:** 2012-01-17

**Authors:** Narges Sharif Razavian, Hetunandan Kamisetty, Christopher J Langmead

**Affiliations:** 1 Language Technologies Institute, Carnegie Mellon University, Pittsburgh, PA 15213, USA; 2 Department of Biochemistry, University of Washington, Seattle, WA 98195, USA; 3 Computer Science Department, Carnegie Mellon University, Pittsburgh, PA 15213, USA; 4 Lane Center for Computational Biology, Carnegie Mellon University, Pittsburgh, PA 15213, USA

## Abstract

We introduce three algorithms for learning generative models of molecular structures from molecular dynamics simulations. The first algorithm learns a Bayesian-optimal undirected probabilistic model over user-specified covariates (e.g., fluctuations, distances, angles, etc). *L*_1 _reg-ularization is used to ensure sparse models and thus reduce the risk of over-fitting the data. The topology of the resulting model reveals important couplings between different parts of the protein, thus aiding in the analysis of molecular motions. The generative nature of the model makes it well-suited to making predictions about the global effects of local structural changes (e.g., the binding of an allosteric regulator). Additionally, the model can be used to sample new conformations. The second algorithm learns a time-varying graphical model where the topology and parameters change smoothly along the trajectory, revealing the conformational sub-states. The last algorithm learns a Markov Chain over undirected graphical models which can be used to study and simulate kinetics. We demonstrate our algorithms on multiple molecular dynamics trajectories.

## Introduction

The three dimensional structures of proteins and other molecules vary in time according to the laws of thermodynamics. Each molecule visits an ensemble of states which can be partitioned into distinct *conformational sub-states *[1,2] consisting of similar structures. The study of these conformational sub-states remains an active area of research [3-5] and has provided valuable insights into biological function, such as enzyme catalysis [5-7] and energy transduction [8].

Molecular dynamics (MD) simulations are often used to characterize conformational dynamics [9]. These simulations are performed by numerically integrating Newton's laws of motion for a set of atoms. Conformational frames are written to disk into a *trajectory *for subsequent analysis. Until recently, MD simulations were limited to time-scales of several tens of nanoseconds (*ns *= 10^-9 ^sec.). Recent advances in hardware and software (e.g., [10-14]) make it possible to investigate conformational dynamics on microsecond (*μs *= 10^-6 ^sec.) and millisecond (*ms *= 10^-3 ^sec.) time-scales. Such long simulations are especially well-suited to identifying and studying the conformational sub-states relevant to biological function. Unfortunately, the corresponding trajectories are often difficult to analyze and interpret due to their size and complexity. Thus, there is a need for algorithms for analyzing such long timescale trajectories. The primary goal of this paper is to introduce new algorithms to do so.

Our approach to analyzing MD data is to learn generative models known as Markov Random Fields (MRF). This is the first time MRFs have been used to model MD data. A MRF is an undirected probabilistic graphical model that encodes the joint probability distribution over a set of user-specified variables. In this paper those variables correspond to the positional fluctuations of the atoms, but the technique can be easily extended to other quantities, such as pairwise distances or angles. The generative nature of the model means that new conformations can be sampled and, perhaps more importantly, that users can make structural alterations to one part of the model (e.g., modeling the binding of a ligand) and then perform inference to predict how the rest of the system will respond.

We present three closely related algorithms. The first algorithm learns a single model from the data. Both the topology and the parameters of the model are learned. The topology of the learnt graph reveals which variables are directly coupled and which correlations are indirect. Alternative methods, such as constructing a covariance matrix cannot distinguish between direct and indirect correlations. Our algorithm is guaranteed to produce an optimal model. Regularization is used to reduce the tendency of over-fitting the data. The second algorithm learns a time-varying model where the topology and parameters of the MRF change smoothly over time. Time-varying models reveal the different conformational sub-states visited by the molecule and the features of the the energy barriers that separate them. The final algorithm learns a Markov Chain over MRFs which can be used to generate new trajectories and study to kinetics.

## Background

### Molecular dynamics simulation

Molecular Dynamics simulations involve integrating Newton's laws of motion for a set of atoms. Briefly, given a set of *n *atomic coordinates $X=\left\{{\vec{X}}_{1},...,{\vec{X}}_{n}:{\vec{X}}_{i}\in {\mathbb{R}}^{3}\right\}$ and the corresponding velocity vectors $V=\left\{{\vec{V}}_{1},...,{\vec{V}}_{n}:{\vec{V}}_{i}\in {\mathbb{R}}^{3}\right\}$, MD updates the positions and velocities of each atom according to an energy potential. The updates are performed via numerical integration, resulting in a conformational *trajectory*. The size of the time step for the numerical integration is normally on the order of a 1-2 femtoseconds (*fs *= 10^-15 ^sec), meaning that a 1 microsecond simulation requires one billion integration steps. In most circumstances, every 1000th to 10000th conformation is written to disc as an ordered series of *frames*.

Traditional methods for analyzing MD data either monitor the dynamics of global statistics (e.g., the radius of gyration, total energy, etc), or else identify sub-states via a clustering the frames [15-17] or through Principal Components Analysis (PCA) and closely related methods (e.g., [18-22]). Clustering based methods do not produce generative models and generally rely on pairwise comparisons between frames and thus run in quadratic time with respect to the number of frames in the trajectory. Our algorithms produce generative models and only perform linear work in the number of frames. This complexity difference is especially important for long timescale simulations. PCA-based methods implicitly assume that the data are drawn from a multivariate Gaussian distribution. Our method makes the same assumption but differs from PCA in two important ways. First, PCA projects the data onto an orthogonal basis. Our method involves no change of basis, making the resulting model easier to interpret. Second, we employ *L*1 regularization when learning the parameters of our model. Regularization is a common strategy for reducing the tendency to over-fit data by, informally, penalizing overly complicated models. We use *L*1 regularization because it has desirable statistical properties. Specifically, it leads to consistent models (that is, given enough data our algorithm learns the true model) while while enjoying high efficiency (that is, the number of samples needed to achieve the true model is small).

More recently, Lange and Grubmüller introduced full correlation analysis [23], which can capture both linear and non-linear correlated motions from MD simulations. The algorithms in this paper are limited to linear models, but we note that they can be easily extended to more complex forms by using non-Gaussian random variables (e.g., [24,25]). Our final algorithm produces models that resemble Markov State Models (MSMs) [26] but are different in that they are fully generative.

### Markov Random Fields

A Markov Random Field M=(G,Θ) consists of an undirected graph G over a set of random variables *X *= {*X*_1_, ..., *X*_*n*_} and a set of functions Θ over the nodes and edges of G. Together, they define the joint distribution *P*(**X**). The topology of the graph determines the set of *conditional independencies *between the variables. In particular, the ith random variable is conditionally independent of the remaining variables, given its neighbors in the graph. Informally, if variables *X*_*i *_and *X*_*j *_are not connected by an edge in the graph, then any correlation between them is indirect. By 'indirect' we mean that the correlation between *X*_*i *_and *X*_*j *_(if any) can be explained in terms of a pathway of correlations (e.g., *X*_*i *_→ *X*_*k *_→ ··· → *X*_*j*_). Conversely, if *X*_*i *_and *X*_*j *_are connected by an edge, then the correlation is direct. Our algorithm automatically detects these conditional independencies and learns the sparsest possible model, subject to fitting the data.

### Gaussian Graphical Models

A *Gaussian Graphical Model *(GGM) or *Gaussian Markov Random Field *is simply a MRF where each variable is normally distributed. Thus, a GGM encodes a multivariate Gaussian distribution. A GGM has parameters $M=\left(\vec{h},\sum^{-1}\right)$ where Σ^-1 ^is an *n *× *n *matrix (known as the *precision matrix*) and $\vec{h}$ is a *n *× 1 vector. The non-zero elements of Σ^-1 ^reveal the edges in the MRF. The inverse of the precision matrix, denoted by Σ, is the covariance matrix for a multivariate Gaussian distribution with mean $\vec{\mu }={\vec{h}}^{T}\sum$.

Gaussian distributions have a number of desirable properties including the availability of analytic expressions for a variety of quantities. For example, the probability of observing $\vec{x}=\left(x_{1},...,x_{n}\right)$ is:

(1) $$P\left(x\right)=\frac{1}{Z}\exp\left\{-\frac{1}{2}{\left(\vec{x}-\vec{\mu }\right)}^{T}\overset{-1}{\sum }\left(\vec{x}-\vec{\mu }\right)\right\},$$

where $Z=\sqrt{{\left(2\pi \right)}^{n}|\sum |}$ is the partition function and |Σ| denotes the determinant of Σ. Other quantities of interest can be computed as well, such as the free energy of the model, - ln *Z*, its differential entropy:

(2) $$\frac{1}{2}\ln\left[{\left(2\pi e\right)}^{n}\right]|\sum |]$$

or the KL-divergence between two different models:

(3) $$KL\left(M_{0}∥M_{1}\right)=1∕2\left(trace\left(\overset{-1}{\underset{1}{\sum }}\underset{0}{\sum }\right)+{\left({\vec{\mu }}_{1}-{\vec{\mu }}_{0}\right)}^{T}\sum_{1}^{-1}\left({\vec{\mu }}_{1}-{\vec{\mu }}_{0}\right)-\ln\left(|\underset{0}{\sum }|∕|\underset{1}{\sum }|\right)-n\right).$$

A GGM can also be used to manipulate a subset of variables and then then compute the marginal densities for the remaining variables. For example, let **V **⊂ **X **be an arbitrary subset of variables **X **and let **W **be the complement set. We can condition the model by setting variables **V **to some particular value, $\vec{v}$. The marginal distribution over **W **given $\vec{v}$ is a multivariate Gaussian with parameters $\left({\vec{\mu }}_{w|\vec{v}},\sum_{W}\right)$ where

(4) $${\vec{\mu }}_{W|\vec{v}}={\vec{\mu }}_{W}+\sum_{W}^{T}\sum_{VV}^{-1}\left(\vec{v}-{\vec{\mu }}_{v}\right)$$

(5) $$\sum_{W}=\sum_{WW}-\sum_{WV}^{T}\sum_{VV}^{-1}\sum_{WV}$$

Here, $\sum =\left(\begin{array}{cc} \sum_{WW} & \sum_{WV}\\ \sum_{WV}^{T} & \sum_{VV} \end{array}\right)$. Thus, inference can be performed analytically via matrix operations. In this way, users can predict the conformational changes induced by local perturbations or, more generally, study the couplings between arbitrarily chosen subsets of variables.

## Algorithms

We now present three algorithms for learning various kinds of generative models from MD data.

**Input **The input to all three algorithms is a time-series of vectors $D=\left({\vec{d}}_{1},...,{\vec{d}}_{t}\right)$ where ${\vec{d}}_{i}$ is a *n *× 1 vector of covariates (e.g., positional and/or angular deviations) and *t *is the number of snapshots in the MD trajectory.

### Algorithm 1

**Output **The first algorithm produces a Gaussian Graphical Model $M=\left(\vec{h},\sum^{-1}\right)$. The first step is to compute the sample mean $\vec{\mu }=1∕t\sum_{i=1}^{t}{\vec{d}}_{i}$. Then it computes the regularized precision matrix Σ^-1 ^(see below). Finally, $\vec{h}$ is computed as follows: $\vec{h}=\sum^{-1}\vec{\mu }$.

The algorithm produces the sparsest precision matrix that still fits the data (see below). It also guarantees that Σ^-1 ^is positive-definite, which means it can be inverted to produce the regularized covariance matrix (as opposed to the sample covariance, which is trivial to compute). This is important because Eqs 1-3 require the covariance matrix, Σ. We further note that a sparse precision matrix does not imply that the corresponding covariance matrix is sparse, nor does a sparse covariance imply that the corresponding precision matrix is sparse. That is, our algorithm isn't equivalent to simply thresholding the sample covariance matrix, and then inverting.

#### Learning regularized precision matrices

A straight-forward way of learning a GGM is to find the parameters ($\left(\vec{\mu },\sum \right)$) that maximize the likelihood of the data (i.e., by finding parameters that maximize $\sum_{i=1}^{t}P\left({\vec{d}}_{i})\right)$. It is known that a maximum likelihood model can be produced by setting the pair $\left(\vec{\mu },\sum \right)$ to the sample mean and covariance matrices, respectively. Unfortunately, maximum likelihood estimates can be prone to over-fitting. This is not surprising because the covariance matrix alone contains *m *= *O*(*n*^2^) parameters, each of which must be estimated from the data. This is relevant because the number of *independent *samples needed to obtain a statistically robust estimate of Σ grows polynomially in *m*. We note that while modern MD simulations do produce large numbers of samples (i.e., frames), these samples are *not *independent (because they form a time-series), and so the effective sample size is much smaller than the number of frames in the trajectory.

Our algorithm addresses the problem of over-fitting by maximizing the following objective function:

(6) $$ll(\sum^{-1}|D)=\sum_{k=1}^{t}\log P({\vec{d}}_{k})-\lambda ∥\sum^{-1}{∥}_{1}.$$

Here, ||Σ^-1^||_1 _is the *L*_1 _norm of the precision matrix. The *L*_1 _norm is defined as the sum of the absolute values of the matrix elements. It can be interpreted as a measure of the complexity of the model. In particular, each non-zero element of Σ^-1 ^corresponds to a parameter in the model and must be estimated from the data. Thus, Eq. 6 establishes a tradeoff between the log likelihood of the data (the first term) and the complexity of the model (the second term). The scalar value λ controls this tradeoff such that higher values produce sparser precision matrices. This is our algorithm's only parameter. Its value can be computed analytically [27] from the number of frames in the trajectory and variables. Alternatively, users may elect to adjust λ to obtain precision matrices of desired sparsity.

Algorithmically, our algorithm maximizes Eq. 6 in an indirect fashion, by defining and then solving a convex optimization problem. Using the functional form of $P\left(\vec{d}\right)$ according to Eq. 1, the log-likelihood of Σ^-1 ^can be rewritten as:

$$ll\left(\sum^{-1}|D\right)=-\log\left(|\sum |\right)-\overset{t}{\underset{k=1}{\sum }}\left({\vec{d}}_{k}-\vec{\mu }\right)\sum^{-1}\left({\vec{d}}_{k}-\vec{\mu }\right)-\lambda ∥\sum^{-1}{∥}_{1}.$$

Noting that $|\sum |=\frac{1}{|\sum^{-1}|}$ and that *trace *(**ABC**) = *trace*(**CAB**), the log-likelihood of Σ^-1 ^can then be rewritten as:

$$ll\left(\sum^{-1}|D\right)=\log\left(|\sum^{-1}|\right)-trace\left(\left(D-\vec{\mu }\right)\sum^{-1}\left(D-\vec{\mu }\right)\right)-\lambda ∥\sum^{-1}{∥}_{1}.$$

Next, using the definition of the sample covariance matrix,

$$S=\langle \left(D-\langle D\rangle \right){\left(D-\langle D\rangle \right)}^{T}\rangle ,$$

we can define the matrix Σ^-1 ^that maximizes 6 as the solution to the following optimization problem:

(7) argmax∑-1≻0 log∣∑-1∣-trace(S∑-1)-λ∥∑-1∥1.

We note that *L*_1 _regularization is equivalent to maximizing the likelihood under a Laplace prior and so the solution to Eq. 7 is a *maximum a posteriori *(MAP) estimate of the true precision matrix, as opposed to a maximum likelihood estimate. That is, our algorithm is a Bayesian method. Moreover, the use of *L*_1 _regularization ensures additional desirable properties including *consistency *-- given enough data, the learning procedure learns the true model, and high statistical *efficiency *-- the number of samples needed to achieve this guarantee is small.

We now show that the optimization problem defined in Eq. 7 is smooth and convex and can therefore be solved optimally. First, we consider the dual form of the objective. To obtain the dual, we first rewrite the *L*_1_-norm as:

$$∥X{∥}_{1}=\underset{∥U{∥}_{\infty }\leq 1}{\max}trace\left(XU\right)$$

where ||**U**||∞ denotes the maximum absolute value element of the matrix **U**. Given this change of formulation, the primal form of the optimization problem can be rewritten as:

(8) max∑-1≻0 min∥U∥∞≤λ log∣∑-1∣-trace(∑-1,S+U).

That is, the optimal Σ^-1 ^is the one that maximizes the worst case log likelihood over all additive perturbations of the covariance matrix.

Next, we exchange the *min *and *max *in Eq. 8. The inner *max *in the resulting function can now be solved analytically by calculating the gradient and setting it to zero. The primal form of the objective can thus be written as:

$$U*=\underset{∥U{∥}_{\infty }\leq \lambda }{\min}-\log|S+U|-n,$$

such that Σ^-1 ^= (**S **+ U*)^-1^.

After one last change of variables, **W **= **S **+ **U**, the dual form of Eq. 7 can now be defined as:

(9) $$\sum *=\max\left\{\log|W|:∥W-S{∥}_{\infty }\leq \lambda \right\}$$

Eq. 9 is smooth and convex, and for small values of *n *it can be solved by standard convex multivariate optimization techniques, such as the interior point method. For larger values of *n *we use Block Coordinate Descent [27] instead.

#### Block Coordinate Descent

Given matrix **A**, let **A**_\*k*\*j *_denote the matrix produced by removing column *k *and row *j *of the matrix. Let **A**_*j *_also denote the column *j*, with diagonal element **A**_*jj *_removed. The Block Coordinate Descent algorithm [27]. Algorithm 1 proceeds by optimizing one row and one column of the variable matrix **W **at a time. The algorithm iteratively optimizes all columns until a convergence criteria is met. The **W**s produced in each iterations are strictly positive definite and so the regularized covariance matrix Σ = *W *is invertible.

**Algorithm 1 **Block Coordinate Descent

**Require**: Tolerance parameter ε, sample covariance **S**, and regularization parameter λ.

   Initialize **W**^(0)^:= **S **+ λ**I **where **I **is the identity matrix.

   **repeat**

      **for ***j *= 1,... *n ***do**

         $y*=\arg\underset{y}{\min}\left\{y^{T}W_{\backslash j\backslash j}^{\left(j-1\right)}y:∥y-S_{j}{∥}_{\infty }\leq \lambda \right\}$ {//Here, **W**^(*j*-1) ^denotes the current iterate.}

         Set **W**^(*j*) ^to **W**^(*j*-1) ^such that **W**_*j *_is replaced by y*.

      **end for**

      Set **W**^(0) ^= **W**^(*n*)^

   **until ***trace*((**W**^(0)^)^-1^**S**) - *n *+ λ||(**W**^(0)^)^-1^||_1 _≤ ε.

   **return W**^(0)^

The time complexity of this algorithm is *O*(*n*^4.5^/ε) [27] when converging to a solution within ε > 0 of the optimal. This complexity is better than $O\left(n^{6}∕\log\left(\frac{1}{\varepsilon }\right)\right)$, which would have been achieved using the interior point method on the dual form [28].

In summary, the algorithm produces a time-averaged model of the data by computing the sample mean and then constructing the optimal regularized Σ by solving Eq. 9 using Block Coordinate Decent. The regularized covariance matrix Σ is guaranteed to be invertible which means we can always compute the precision matrix, Σ^-1^, which can be interpreted as a graph over the variables revealing the direct and indirect correlations between the variables.

### Algorithm 2

The second algorithm is a straight-forward extension of the first. Instead of producing a time-averaged model, it produced time-varying model: $M\left(\tau \right)=\left(\vec{h}\left(\tau \right),\sum^{-1}\left(\tau \right)\right)$. Here, *τ *≤ *t *indexes over sequentially ordered windows of frames in the trajectory. The width of the window, *w*, is a parameter and may be adjusted to learn time-varying models at a particular time-scale. Naturally, a separate time-averaged model could be learned for each window. Instead, the second algorithm applies a simple smoothing kernel so that the parameters of the *τ*th window includes information from neighboring window too. In this way, the algorithm ensures that the parameters of the time-varying model evolve as smoothly as possible, subject to fitting the data.

Let **D**^(*τ*) ^⊆ **D **denote the subset of frames in the MD trajectory that correspond to the τth window, 1 ≤ *τ *≤ *T*. The second algorithm solves the following optimization problem for each 1 ≤ *τ *≤ *T*:

∑-1(τ)= argmaxX≻0 log∣X∣-trace(S(τ)X)-λ∥X∥1

Here, S(*τ*) is the *weighted covariance matrix*, and is calculated as follows:

$$S\left(\tau \right)=\frac{\sum_{k=\tau -\kappa }^{\tau +\kappa }w_{k}\langle \left(D^{\left(k\right)}-\langle D^{\left(k\right)}\rangle \right){\left(D^{\left(k\right)}-\langle D^{\left(k\right)}\rangle \right)}^{T}\rangle }{\sum_{k=\tau -\kappa }^{\tau +\kappa }w_{k}}$$

where *k *indexes over windows *τ *- *κ *to *τ *+ *κ*, *κ *is a user-specified kernel width, and the weights *w*_*k *_are defined by a nonnegative kernel function. The choice of kernel function is specified by the user. In our experiments the kernel mixed the current window and the previous window with the current window having twice the weight of the previous. The time-varying model is then constructed by solving Eq. 9 for each **S**(*τ*). That is, the primary difference between the time-averaged and time-varying version of the algorithm is the kernel function.

### Algorithm 3

The final algorithm builds on the second algorithm. Recall that the second algorithm learns *T *sequentially ordered models over windows of the trajectory. Moreover, recall that each model encodes a multivariate Gaussian (Eq. 1) and that the KL-divergence between multivariate Gaussians can be computed analytically via Eq. 3. The KL-divergence (also known as information gain or relative entropy) is a non-negative measure of the difference between two probability distributions. It is zero if and only if the two distributions are identical. It is not, however, a distance metric because it is not symmetric. That is *D*(*P*||*Q*) ≠ *D*(*Q*||*P*), in general. However, it is common to define a symmetric KL-divergence by simply summing *KL*_*sym *_= *D*(*P*||*Q*)+*D*(*Q*||*P*). We can thus cluster the models using any standard clustering algorithm, such as k-means or a hierarchial approach. In our experiments we used complete linkage clustering, an agglomerative method that minimizes the maximum distance between elements when merging clusters.

Let *S *be the set of clusters returned by a clustering algorithm. Our final algorithm treats those clusters as states in a Markov Chain. The prior probability of being in each state can be estimated using free energy calculations [29,30] for each cluster, or according to the relative sizes of each cluster. It then estimates the transition probabilities between states *i *and *j *by counting the number of times a model assigned to cluster *i *is followed by a model assigned to cluster *j*. This simple approach creates a model that can be used to generate new trajectories by first sampling states from the Markov Chain and then sampling conformations from the models associated with that state.

## Experiments

We applied our algorithms to several molecular dynamics simulation trajectories. In this section, we illustrate some of the results obtained through this analysis. The algorithms were implemented in Matlab and run on a dual core T9600 Intel processor running at 2.8 Ghz. The wall-clock runtimes for all the experiments were on the order of seconds to about 10 minutes, depending on the size of the data set and parameter settings.

### Algorithm 1: application to the early events of HIV entry

**Figure 1:**
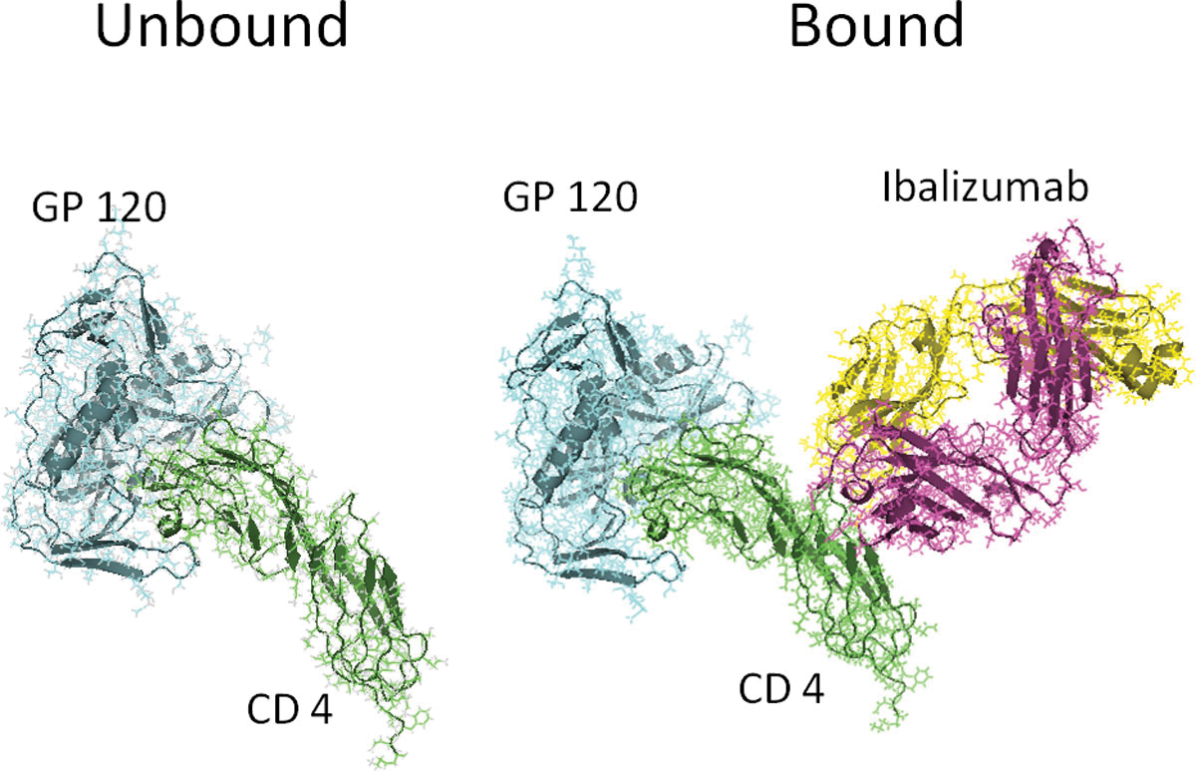
(Left) gp120 (blue) bound to CD4 (green). (Right) The same complex bound to Ibalizumab (yellow and purple), a monoclonal antibody HIV entry inhibitor. Notice that Ibalizumab does not bind to gp120.

We applied the first algorithm to simulations of a complex (Figure 1-left) consisting of gp120 (a glycoprotein on the surface of the HIV envelope) and the CD4 receptor (a glycoprotein expressed on the surface of T helper cells). The binding of gp120 to CD4 receptors is among the first events involved in HIV's entry into helper T-Cells. We performed two simulations using namd [31]. The first simulation was the gp120-CD4 complex in explicit solvent at 310 degrees Kelvin. The second simulation was the same complex bound to Ibalizumab (Figure 1-right), a humanized monoclonal antibody that binds to CD4 and inhibits the viral entry process [32]. Each trajectory was each 2 ns long and contained 4500 frames.

Ibalizumab's mechanism of action is poorly understood. As can be seen in Figure 1, Ibalizumab does not prevent gp120 from binding to CD4, nor does it directly bind to gp120 itself, suggesting that its inhibitory action occurs via an allosteric mechanism. To investigate this phenomenon, we applied our first algorithm to the two trajectories and then compared the resulting models. The variables in the models corresponded to the positional fluctuations of the C-*α *atoms, relative to the initial frame of the simulation.

#### Correlation networks

**Figure 2:**
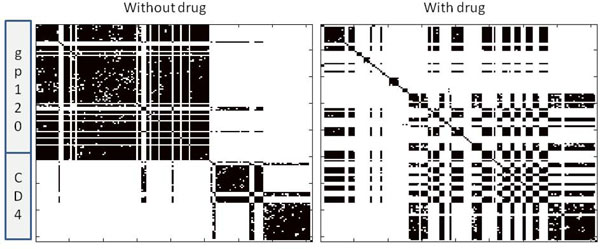
**gp120-CD4 correlation networks learned with Algorithm 1.** (Left) Edges learned by algorithm for the drug-free simulation. (Right) Edges learned by algorithm for the drug-bound simulation.

Figure 2 illustrates the correlation networks learned from the drug-free (left) and drug-bound (right) simulations. The same lambda value (250) was used in each case. In each panel, a black dot indicates that residue *i *is connected to residue *j *in the graphical model. The residues corresponding to gp120 and CD4 are labeled on the left-hand side. Edges exist between both spatially proximal and distant residues. For these panels, only the data from the gp120 and CD4 atoms were modeled. However, the effects of the drug are obvious. In the drug-free case the direct correlations are largely intra-molecular, with inter-molecular correlations limited to the binding interface. The drug-bound model, in contrast, exhibits many more inter-molecular edges. Moreover, the drug-bound gp120 has far fewer inter-molecular edges. That is, Ibalizumab not only modulates the interactions between gp120 and CD4, it also changes the internal correlation structure of gp120, despite the fact that the drug only binds to CD4. This is consistent with the hypothesis that Ibalizumab's inhibitory action occurs via an allosteric mechanism.

The probabilistic nature of the model means that it is possible to compute the likelihood of each data set under both models. Table 1 presents the log-likelihoods of both data sets under both models. As expected, the log-likelihood of the unbound data is larger (i.e., more likely) under the unbound model than it is under the bound model, and visa-versa. That is, the models are capturing statistical differences between the simulations.

**Table 1:** Log-likelihood (LL) of the gp120-CD simulations under both models

*Data*	LL**(*Data*|*Unbound Model*)**	LL**(*Data*|*Drug *- *Bound Model*)**
Unbound	-0.03	-0.19
Bound	-0.04	-0.29

**Figure 3:**
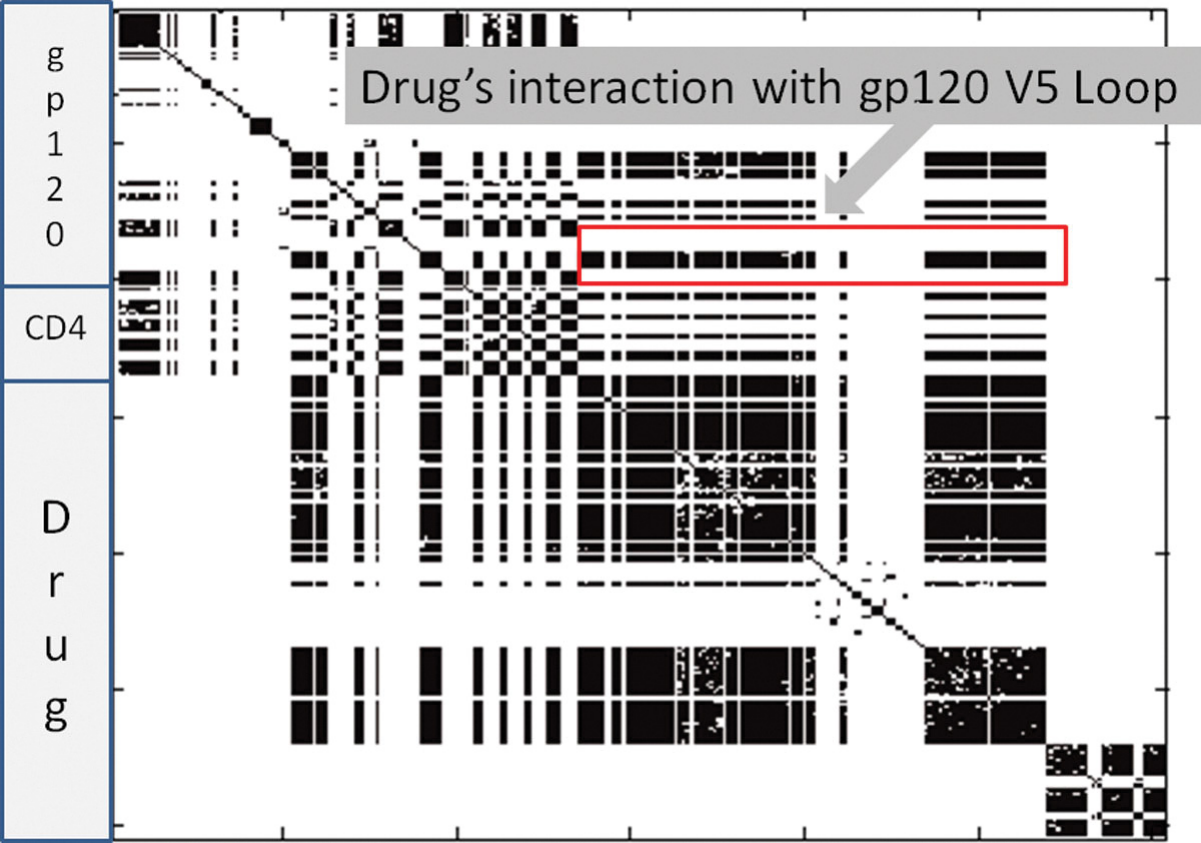
**gp120-CD4-Ibalizumab correlation networks learned with Algorithm 1.** Edges learned by algorithm for the drug-bound simulation. Here, all three models are shown.

Figure 3 illustrates the correlation networks learned for all three molecules in the drug-bound simulation. A red box encompasses edges between the drug and the V5 loop of gp120. These particular couplings are interesting because it is known that mutations to the V5 loop can cause resistance to Ibalizumab [33]. Future simulations of such mutants might provide further insights into the mechanism of resistance.

#### Comparison to sub-optimal models

**Figure 4:**
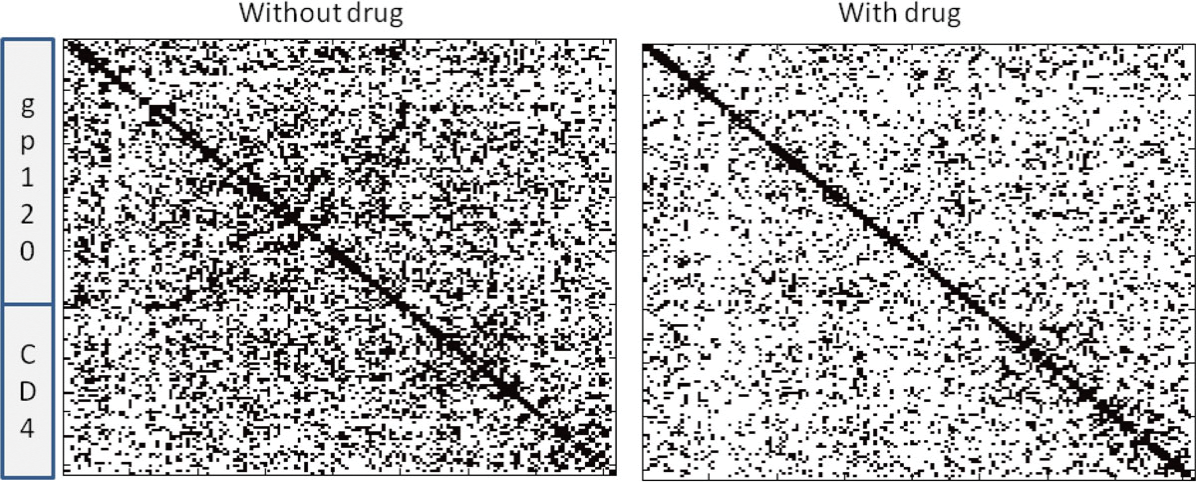
**Thresholded precision matrix models.** (Left) Edges produced by thresholding inverse of sample covariance matrix for the drug-free simulation. (Right) Edges produced by thresholding inverse of sample covariance matrix for the drug-bound simulation. Notice that the edges lack the kind of structure seen in Figures 2 and 3.

Our method is guaranteed to return an optimal model. Here we compare the models returned by our algorithm to those obtained by a reasonable, but nevertheless sub-optimal algorithm for generating sparse networks. For comparison, we inverted the *sample *covariance matrices for each data set. The resulting sample precision matrices were then thresholded so that they had the same number of edges as the ones produced via our method. We find that while the resulting models have similar fits to the data (-0.02 log-likelihood for the unbound trajectory; -0.03 log-likelihood for the bound trajectory), the *L*_1 _penalty is is much larger in each case (0.86 vs 15.1 for unbound; 0.75 vs 12.9 for bound). The difference in *L*_1 _penalties is due to the radically different choices of edges each method makes. Only 41% (resp. 31%) of the unbound (resp. bound) edges match the ones identified by our algorithm. Moreover, the thresholded sample precision matrices (Figure 4) lack the kind of structure seen in Figure 2. Thus, in addition to producing models that maximize Eq. 6, the resulting models are potentially easier to interpret.

#### Perturbation analysis

**Figure 5:**
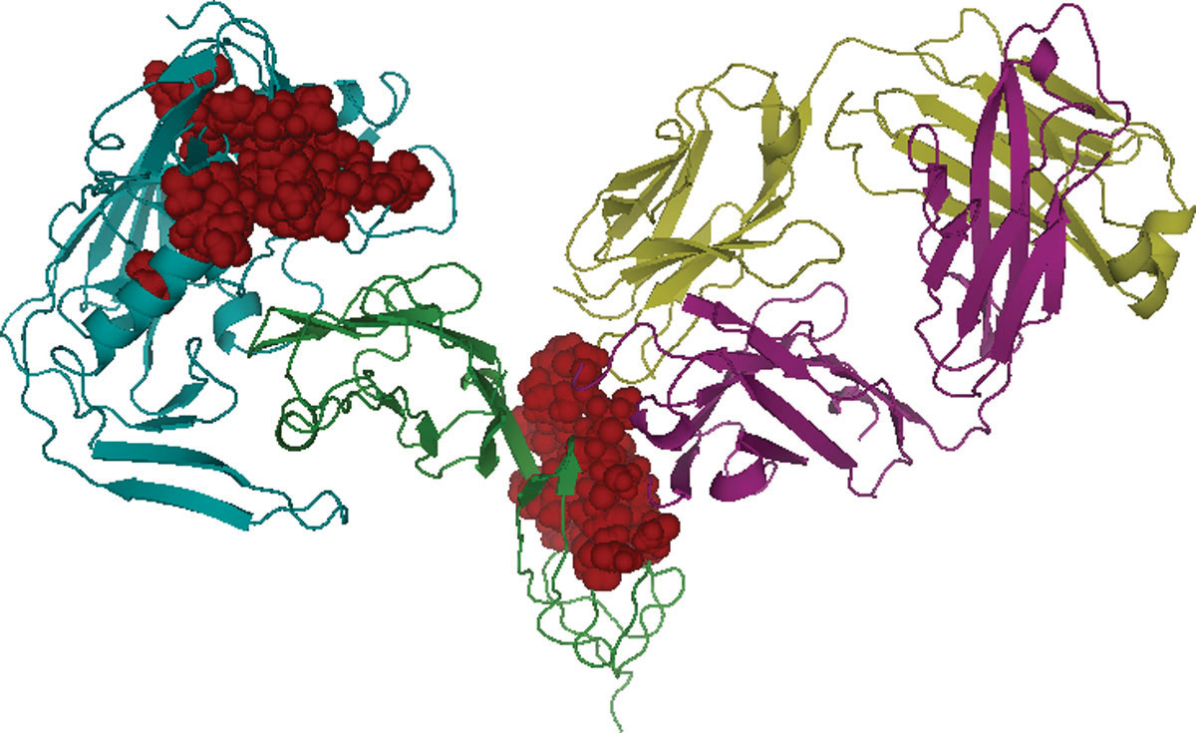
**Sensitivity to perturbations.** The red spheres mark the residues that are most sensitive to perturbations in the drug.

Next, we demonstrate the use of inference to quantify the sensitivity of gp120 to structural perturbations in the drug. We conditioned the model learned from the trajectory with gp120, CD4 and Ibalizumab on the structure of the drug and then performed inference (Eq. 4) to compute the most likely configuration of remaining variables (i.e., those corresponding to gp120 and CD4). This was repeated for each frame in the trajectory. The residues with the highest average displacement are illustrated as red spheres in Figure 5. As expected, the residues that form the binding interface between CD4 and Ibalizumab are sensitive Ibalizumab's motions. Interestingly, a number of gp120 residues are also sensitive, including residues in the vicinity of the V5 loop.

### Algorithm 2: application to a 1 microsecond simulation of the engrailed homeodomain

**Figure 6:**
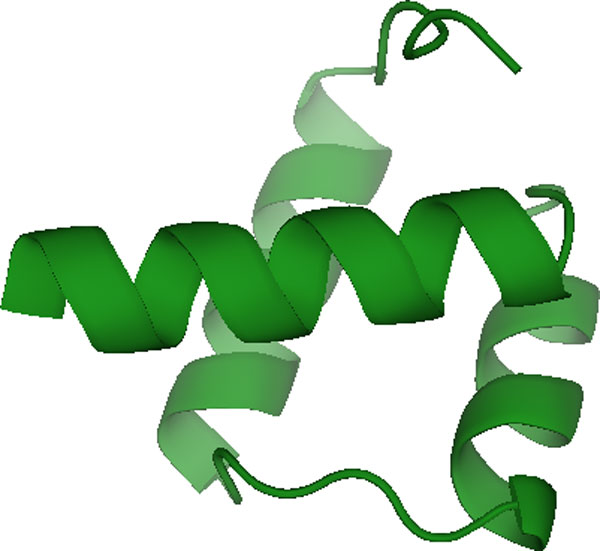
Engrailed homeodomain.

We applied the second algorithm to a simulation of the engrailed homeodomain (Figure 6), a 54-residue DNA binding domain. The DNA-binding domains of the homeotic proteins, called homeodomains (HD), play an important role in the development of all metazoans [34] and certain mutations to HDs are known to cause disease in humans [35]. Homeodomains fold into a highly conserved structure consisting of three alpha-helices wherein the C-terminal helix makes sequence-specific contacts in the major groove of DNA [36]. The Engrailed Homeodomain (En-HD) is an ultra-fast folding protein that is predicted to exhibit significant amounts of helical structure in the denatured state ensemble [37]. Moreover, the experimentally determined unfolding rate is of 1.1E + 03/sec [38], which is also fast. Taken together, these observations suggest that the protein may exhibit substantial conformational fluctuations at equilibrium.

We performed three 50-microsecond simulations of the protein at 300, 330, and 350 degrees Kelvin. These simulations were performed on ANTON[14], a special-purpose supercomputer designed to perform long-timescale simulations. Each simulation had more than 500,000 frames. In this paper, we learned a time-varying model of the first microsecond of the 300 degree trajectory, modeling the fluctuations of the alpha carbons. The window size was 2 ns, and a sawtooth smoothing kernel was applied such that the *i*th model is built from the data from windows *i*, *i *-1, and *i *- 2 such with kernel weights 0.57, 0.29, and 0.14, respectively. A total of 500 models were learned from the first microsecond of the trajectory.

**Figure 7:**
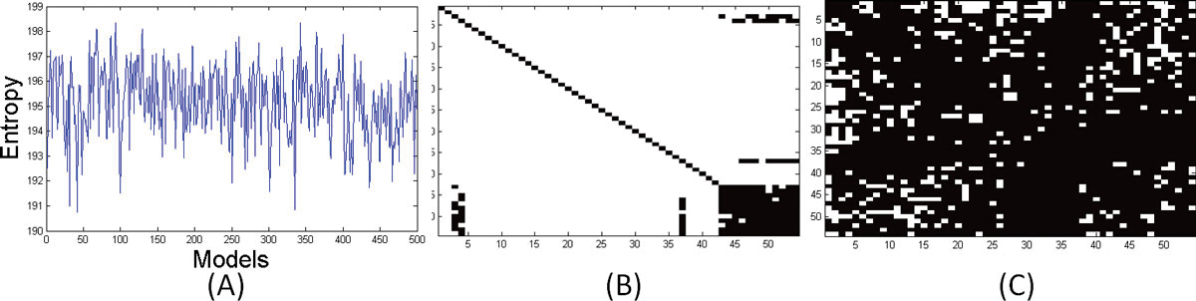
(A) Differential entropy of the 500 model learned from the engrailed trajectory. (B) Correlation network of the model with the smallest differential entropy (model 42). (C) Correlation network of the model with the largest differential entropy (model 342).

Figure 7-A plots the differential entropy (Eq. 2) of the 500 models. We see that the curve has a variety of peaks and valleys that can be used to segment the trajectory into putative sub-states. Figures 7-B and 7-C illustrate the correlation networks obtained from the models with the smallest and largest differential entropies, respectively. As can be seen, the simulation visits sub-states that have radically different correlation structures.

**Figure 8:**
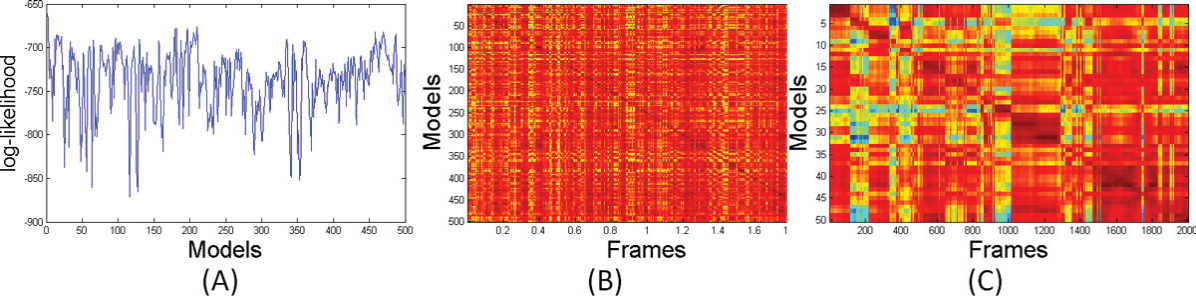
(A) Average log-likelihood of the frames from the *i *+ 1st window under the ith model. Sudden drops in likelihood mark the transition between sub-states. (B) Log-likelihoods for each frame under each of the 500 models. (C) The first 50 rows and first 2,000 columns of the matrix from panel B. The block-structure illustrates the sub-states visited in the first 2,000 frames.

Figure 8-A plots the average log-likelihood of the frames from the *i *+ 1st window under the ith model. Sharp drops in the likelihood can also be used to segment the trajectory into possible sub-states and to pin-point the moment when the system transitions between them. Figure 8-B shows the log-likelihood of each of the frames under each of the 500 models. Figure 8-C shows the first 50 rows and the first 2,000 columns of Figure 8-B. The clear block-structure of the matrix more clearly illustrates the sub-states visited by the simulation.

**Figure 9:**
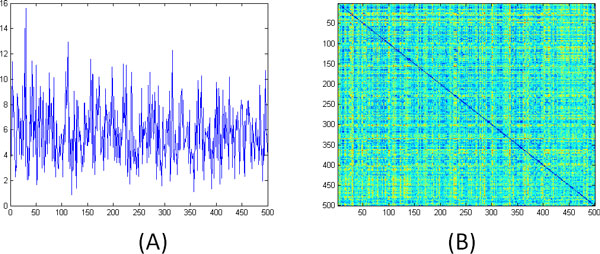
(A) KL-divergence between sequential models. (B) Pairwise KL-divergences between models.

Figure 9-A plots the symmetric version of the KL-divergence (Eq. 3) between sequential models. Once again, spikes in this curve can be used to segment the trajectory.

### Algorithm 3: application to a 1 microsecond simulation of the engrailed homeodomain

Using the 500 models learned in the previous section, we computed the symmetric KL-divergence between all pairs of models. Recall that the KL-divergence (Eq. 3) is a measure of the difference between distributions. Figure 9-B plots the pairwise KL divergences between the 500 models.

**Figure 10:**
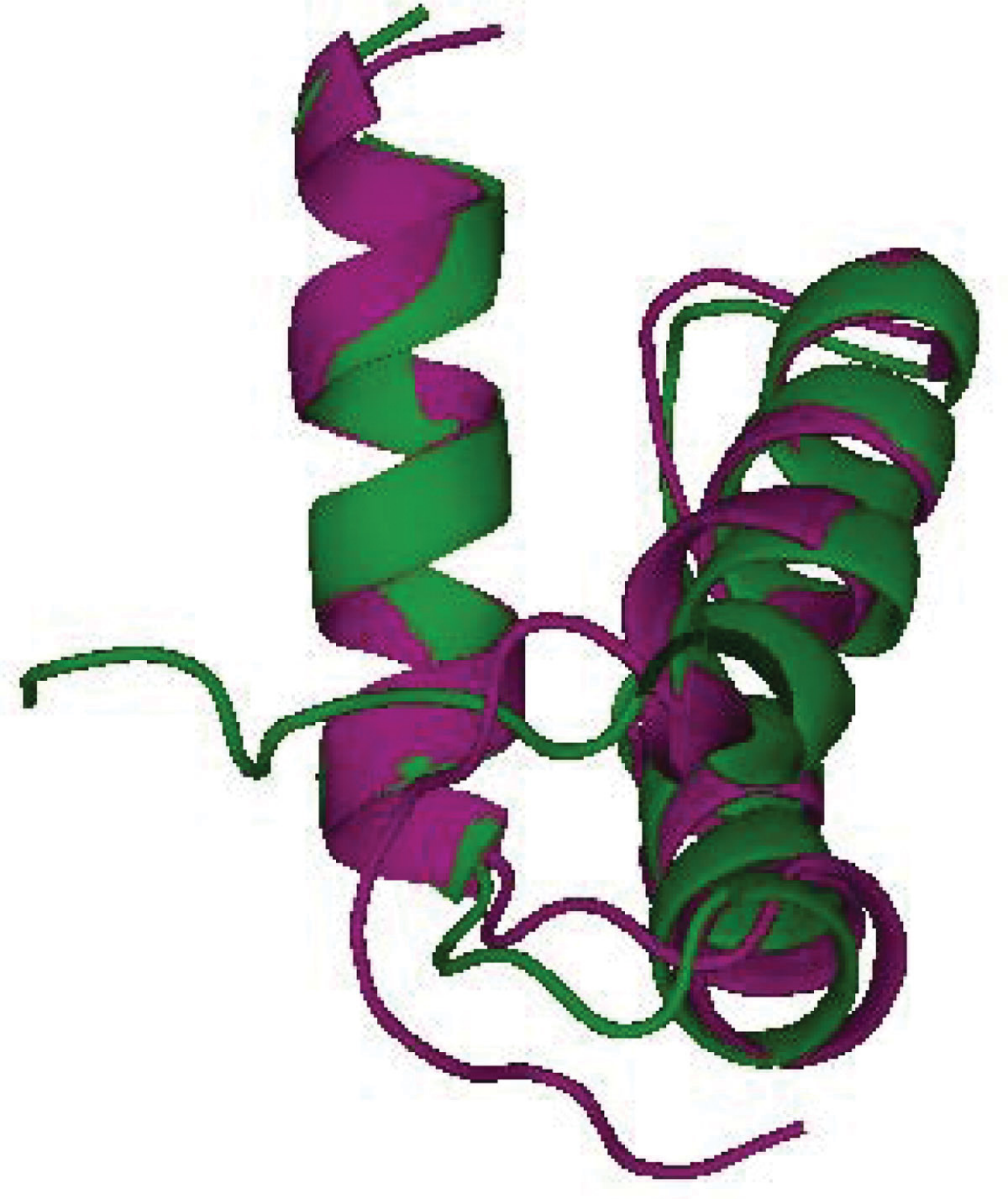
Representative structures for states 4 (green) and 6 (magenta).

We then applied complete linkage clustering to the KL-divergence matrix. Complete linkage clustering minimizes the maximum distance between elements when merging clusters. We selected a total of 7 clusters based on the assumption that the number of sub-states visited by a sequence of *m *models proportional to the logarithm of *m*. The intuition behind this assumption is that different sub-states are separated by energy barriers and the probability of surmounting an energy barrier is exponentially small in the height of the barrier. Figure 10 shows two representative structures from the two largest clusters. As can be seen, the primary difference between the two structures is the N-terminal loop.

**Figure 11:**
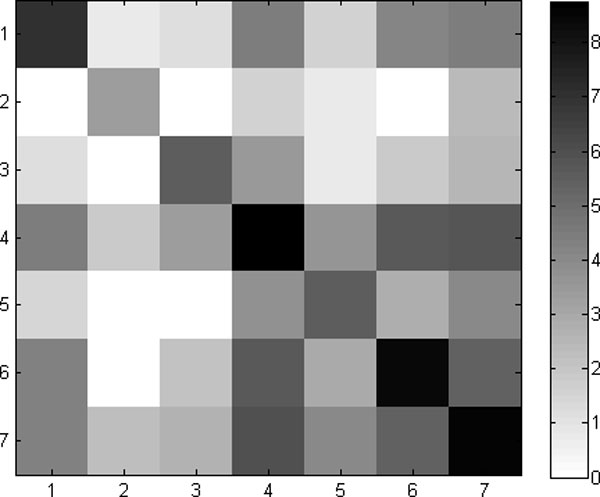
**State-transition matrix.** The color indicates the log of the number of times state *i *transitions to state *j*.

Finally, we estimated the parameters of a Markov chain over the 7 clusters by counting the number of times a model from the *i*th cluster was followed by a model from the *j*th cluster. The resulting state-transition matrix is shown in Figure 11. The matrix indicates that state 4 is the dominant state, but inter-converts with states 6 and 7. This state-transition matrix and the graphical models associated with each state encapsulate the statistics of the trajectory.

## Discussion

Many existing techniques for analyzing MD data are closely related to, or direct applications of Principal Components Analysis (PCA). Quasi-Harmonic Analysis (QHA) [18,19], for example, is PCA applied to a mass-weighted covariance matrix of atomic fluctuations. PCA-based methods diagonalize the covariance matrix and thus produce a set of eigenvectors and corresponding eigenvalues. Each eigenvector can be interpreted as one of the principal modes of vibration within the system or, equivalently, as a normally distributed random variable with zero mean and variance proportional to the corresponding eigenvalue. That is, PCA-based methods model the data in terms of a multivariate Gaussian distribution. Our methods also build multivariate Gaussian models of the data but does so over the real-space variables, not the eigen-space variables.

PCA-based methods generally project the data onto a low-dimensional subspace spanned by the eigenvectors corresponding to the largest eigenvalues. This is done to simplify the data and because lower dimensional models tend to be more robust (i.e., less likely to over-fit the data). Our methods, in contrast, uses regularization when estimating the parameters of the model to achieve the same goals.

The eigenvectors produced by PCA-based methods contain useful information about how different regions of the system move in a coordinated fashion. In particular, the components of each vector quantify the degree of coupling between the covariates in that mode. However, the eigenvectors make no distinction between direct and indirect couplings. Moreover, eigenvectors are an inherently global description of dynamics. Our methods, in contrast, do not perform a change of basis and instead models the data in terms of a network of correlations. The resulting model, therefore, reveals which correlations are direct and which are indirect. Pathways in these networks may provide mechanistic insights into important phenomena, such as allosteric regulation. Our models can also be used to investigate motions that are localized to specific regions of the system.

Finally, we note that because our first algorithm produces a regularized estimate of the true covariance matrix, Σ, it could potentially be used as a pre-processing step for PCA-based methods, which normally take as input the sample covariance matrix.

## Conclusions and future work

We have introduced three novel methods for analyzing Molecular Dynamics simulation data. Our algorithms learn regularized graphical models of the data which can then be used to: (i) investigate the networks of correlations in the data; (ii) sample novel configurations; or (iii) perform *in silico *perturbation studies. We note that our methods are complementary to existing analysis techniques, and are not intended to replace them.

There are a number of important areas for future research. Gaussian Graphical Models have a number of limitations, most notably that they encode uni-modal distributions and are best suited to modeling harmonic motions. Boltzmann distributions, in contrast, are usually multi-modal. Our third algorithm partially addresses this problem by creating a Markov chain over GGMs but the motions are still harmonic. Discrete distributions could be used to model anharmonic motions (e.g., by adapting the algorithm in [24]). Gaussian distributions are also best suited to modeling variables defined on the real-line. Angular variables, naturally, are best modeled with circular distributions, like the von Mises. We've recently developed an algorithm for learning multivariate von Mises graphical models [25] which could be used to model distributions over bond and dihedral angles.

## List of abbreviations used

GGM: Gaussian Graphical Model; KL: Kullback Leibler; MAP: maximum a posteriori; MD: Molecular dynamics; MRF: Markov Random Field; MSM: Markov State Model; PCA: Principal Components Analysis; QHA: Quasi-Harmonic Analysis.

## Competing interests

The authors declare that they have no competing interests.

## Authors' contributions

All three authors contributed to the creation and implementation of the algorithms and writing the manuscript. N.S.R. and C.J.L. performed the experiments and analysis.
